# Relationship between plasma anti-Müllerian hormone concentrations in female Holstein calves immediately after birth and milk yield and composition in dams during early to mid gestation

**DOI:** 10.3168/jdsc.2024-0543

**Published:** 2024-03-29

**Authors:** Saki Morimatsu, Nagisa Nagami, Chiho Kawashima

**Affiliations:** Field Center of Animal Science and Agriculture, Obihiro University of Agriculture and Veterinary Medicine, Obihiro, Hokkaido, 080-8555, Japan

## Abstract

The energy status of dams during the early stages of gestation may affect ovarian reserves. It was hypothesized that greater milk yield and composition during the early stages of gestation may suppress the number of primordial follicles in the fetuses of dairy cattle. This study aimed to evaluate the anti-Müllerian hormone (AMH), used as an indicator of ovarian reserves, in female Holstein calves immediately after birth. It also examined the relationship between AMH concentrations and the milk yield and composition of dams during early pregnancy. A total of 85 female Holstein calves (born to first-calving cows [nulliparous heifers], n = 31; second-calving [primiparous] cows, n = 22; and third- or subsequent-calving [multiparous] cows, n = 32) were examined. Blood samples were collected from the female calves after birth and before the first colostrum feeding for the analysis of plasma AMH concentrations. Data on daily milk yield and monthly milk composition during the first to sixth month of pregnancy were additionally collected from milking dams. The plasma AMH concentrations ranged from 0.106 to 1,542.3 pg/mL, with an average of 291.6 ± 30.5 pg/mL. No significant differences were observed among the plasma AMH concentrations in female calves born from nulliparous heifers, primiparous cows, or multiparous cows. Primiparous cows exhibited numerous negative correlations between the plasma AMH concentrations of their female calves immediately after birth and the daily milk yield and milk composition from the first to the fourth month of pregnancy; however, no correlation was observed in multiparous cows. Overall, large individual variations were observed in the plasma AMH concentrations immediately after birth. Both milk yield and the amount of milk components strongly influenced plasma AMH concentrations in calves immediately after birth in primiparous cows in the growth phase. These factors, conversely, did not affect the plasma AMH concentrations of calves in multiparous cows. The results of this study suggest that high-producing pregnant primiparous cows devote more energy to their growth and lactation, potentially compromising the reproductive potential of their offspring. Thus, improving the feeding management of primiparous cows with the goal of increasing the fertility of their progeny will help high-yielding dairy cows reproduce more successfully.

## Highlights

• Large individual variations were observed in the AMH of calves.
• Large individual variations reflect energy status and adaptation in dams.
• High milk yield in primiparous cows reduces the AMH concentrations of offspring.
• Milk yield in multiparous cows does not affect the AMH concentrations of offspring.

## Graphical Abstract

**Figure:**
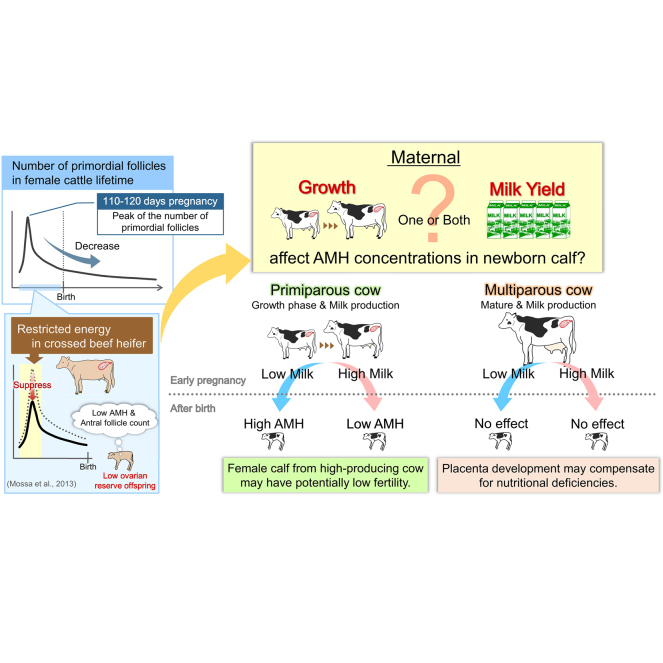
**Summary:** This study investigated the anti-Müllerian hormone (AMH), used as an indicator of ovarian reserves, in female Holstein calves immediately after birth and examined the relationship between AMH concentration and milk yield and the composition of dams during early pregnancy. Large individual variations were observed in plasma AMH concentrations in female calves immediately after birth and before the first colostrum feeding (0.106-1,542.3 pg/mL). Both milk yield and the number of milk components during early pregnancy strongly influenced the plasma AMH concentrations of offspring in primiparous cows in the growth phase. Conversely, the development of the uterine artery and placenta may compensate for nutritional deficiencies in the fetus in multiparous cows; these factors, however, do not affect the plasma AMH concentrations of their calves.

Anti-Müllerian hormone (**AMH**) is a dimeric glycoprotein of 140 kDa from the transforming growth factor β family (La Marca and Volpe, 2006). Anti-Müllerian hormone is primarily secreted by the granulosa cells of healthy growing follicles (La Marca and Volpe, 2006) and plays a role in inhibiting primordial follicular growth from the primordial follicle reserve to avoid premature exhaustion of the ovarian follicular reserve (Dewailly et al., 2014). It additionally modulates follicular development by reducing the responsiveness to follicle-stimulating hormones of preantral and small antral follicles (Dewailly et al., 2014). Moreover, AMH is highly positively associated with antral follicle count (Ireland et al., 2008) and, therefore, has been used as an indicator of ovarian reserves and potential reproductive longevity (Ireland et al., 2008; Rico et al., 2009).

Follicles are present in bovine fetal ovaries during early stages of gestation, with the number of primordial follicles increasing up to d 110 to 120 (Erickson, 1966; Tanaka et al., 2001). After a peak in the number of these follicles, they either do not markedly change until birth (Tanaka et al., 2001) or decline abruptly toward the end of pregnancy (Erickson, 1966). Mossa et al. (2013) demonstrated that the offspring of dams fed with a 60% restricted energy requirement for maintenance during the first trimester of gestation exhibited lower serum AMH concentrations and antral follicle counts during the growth period than those fed a 120% energy requirement. In other words, a dam's energy level during the early stages of gestation may affect the ovarian reserve, and in turn, the potential reproductive longevity of their offspring.

Primiparous and multiparous Holstein high-producing dairy cows yield approximately 30 and 35 kg of milk per day, respectively (Livestock Improvement Association of Japan, https://liaj.lin.gr.jp/wp-content/uploads/2024/03/R03matome.pdf, 2021), during the early stages of gestation when there is an increase in the number of primordial follicles in the fetus. Furthermore, in addition to allocating ingested nutrients for lactation, primiparous cows also use them for growth, vital maintenance, body fat accumulation, and placental and fetal development during pregnancy (Redmer et al., 2004; National Agriculture and Food Research Organization, 2017). Previous studies comparing AMH concentrations among parities have shown that primiparous cows have lower AMH concentrations than multiparous cows with a parity of 2 or 3 (Gobikrushanth et al., 2018, 2019). Moreover, female Holstein cattle have lower blood AMH concentrations than female beef cattle (Hirayama et al., 2017; Mossa et al., 2017) and female Jersey cattle (Ribeiro et al., 2014; Gobikrushanth et al., 2019), which produce less milk than Holstein cows. Schwarzmann et al. (2023) found milk yield was lower in multiparous cows with high AMH concentrations than in cows with low AMH concentrations. It was, therefore, hypothesized that one of the reasons high-producing dairy cows have lower reproductive performance is that higher milk yield, milk components, and growth in primiparous cows during the early stages of gestation may suppress the AMH concentrations of offspring. However, only a few studies to date have investigated the relationship between the AMH concentrations of offspring and milk yield of dams. Succu et al. (2020) found that heifers born to dams that were not being milked during gestation had lower AMH concentrations compared with the offspring of cows in their first lactation. Additionally, the antral follicle count of offspring was positively associated with dam milk fat concentration and milk fat-to-protein ratio of dams during pregnancy (Walsh et al., 2014). However, most studies examining blood AMH concentrations were conducted on cattle aged at least 2 mo or older. Mossa et al. (2017) determined that circulating AMH concentrations increased during the first 2 mo of age, decreased at 5 mo of age, and then stabilized at 8 to 9 mo of age around puberty. The study concluded that variations in AMH concentrations observed before puberty reflect changes in the growth patterns of the small antral follicles and alterations in the ability of granulosa cells to secrete AMH. Namely, the nutritional status of offspring also influences AMH concentrations after birth. Blood AMH concentrations in newborn calves immediately after parturition are, therefore, considered appropriate for assessing the ovarian reserve acquired during the fetal period. However, no studies have investigated blood AMH concentrations in newborn female calves. Therefore, this study investigated AMH concentrations in female Holstein calves immediately after birth and before feeding colostrum. It also examined the relationship between AMH concentrations and the milk yield and composition of dams during early pregnancy.

The experimental procedures in the present study were compiled using the Guide for the Care and Use of Agricultural Animals of Obihiro University (approval number: #21–155). A total of 85 female Holstein calves (born to first-calving cows [nulliparous heifers], n = 31; second-calving [primiparous] cows, n = 22; and third- or subsequent-calving [multiparous] cows, n = 32) were born between December 2018 and September 2022. Blood samples of the female calves were collected from the jugular vein immediately after birth and before the first colostrum feeding using sterile 10-mL tubes containing 200 µL of stabilizer solution (0.3 *M* EDTA, 1% acetylsalicylic acid, pH 7.4) for the analysis of plasma AMH concentrations. The female calves were cleaned, dried with a towel, and weighed before the first colostrum feeding. The tubes were then centrifuged at 2,000 × *g* for 15 min at 4°C, and the plasma samples were stored at −30°C until analyses. The concentrations of plasma AMH were determined using an ELISA kit (Bovine AMH ELISA AL-114; Ansh Labs, Webster, TX). The mean intra- and interassay CV were found to be 2.6% and 5.8%, respectively. Data on daily milk yield and monthly milk composition were additionally collected during the first to sixth month of pregnancy from dams without mastitis (primiparous cows, n = 22; multiparous cows, n = 32). The relationship between plasma AMH concentrations of the female calves and the birth BW or milk yield and milk composition of their dams was then analyzed using Pearson correlation or Spearman rank correlation analysis after statistical testing for normality using the Shapiro–Wilk test (SigmaPlot 13; Systat Software Inc., San Jose, CA). Moreover, the data were analyzed using a one-way ANOVA (SigmaPlot) to compare plasma AMH concentrations of female calves among the parity of their dams. The results are reported as the mean ± SEM; *P <* 0.05 was considered significant.

Figure 1 presents a histogram of the plasma AMH concentrations in female calves immediately after birth and before the first colostrum feeding. The plasma AMH concentrations were found to range from 0.106 to 1,542.3 pg/mL, with an average value of 291.6 ± 30.5 pg/mL and a median of 194.9 pg/mL. The plasma AMH concentrations of female calves born from nulliparous heifers, primiparous cows, and multiparous cows were found to be 252.0 ± 48.5, 258.8 ± 42.8, and 352.4 ± 58.6 pg/mL, respectively, with no differences among the 3 groups (all, *P* > 0.1). In addition, although there was a significant difference in the birth BW of female calves (nulliparous heifers, 41.2 ± 0.7; primiparous cows, 44.4 ± 1.3; and multiparous cows, 44.6 ± 0.9 kg) among the 3 groups (nulliparous heifers vs. primiparous cows, *P* = 0.007; nulliparous heifers vs. multiparous cows, *P* = 0.013; primiparous cows vs. multiparous cows, *P* > 0.1), no correlation was observed between the plasma AMH concentrations and birth BW (r = −0.034, *P* = 0.759).

**Figure 1:**
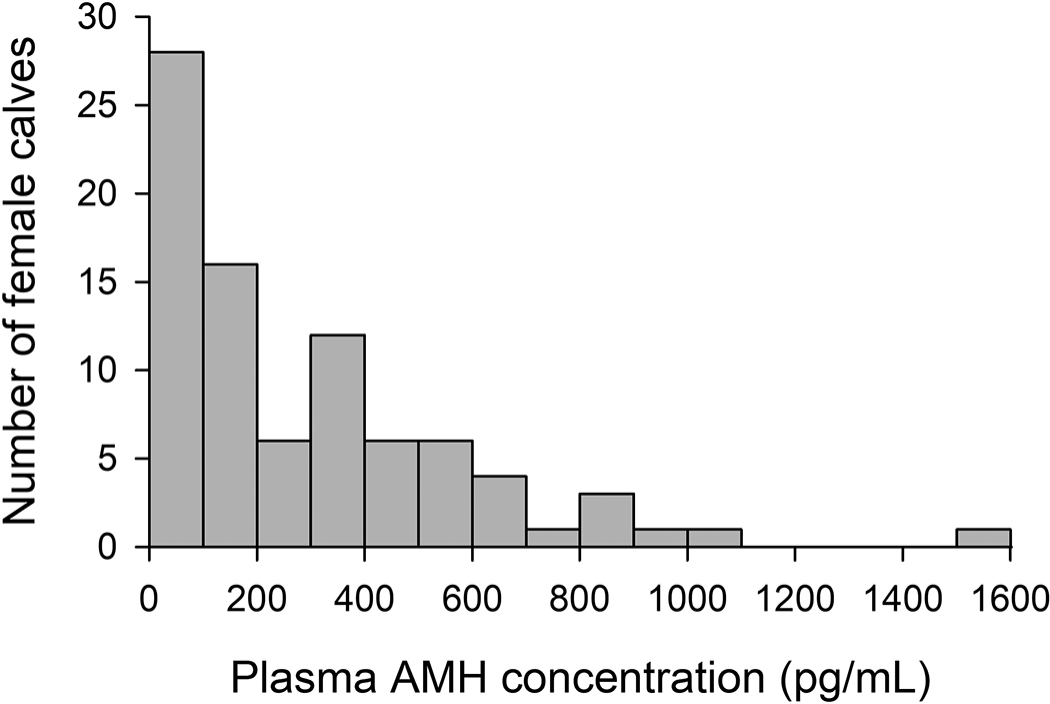
Histogram of the frequency distribution of plasma AMH concentrations in female Holstein calves immediately after birth and before first colostrum feeding (n = 85).

Table 1 presents the correlation coefficient between plasma AMH concentrations in female calves and milk yield or milk composition of their dams (primiparous cows) during the first to sixth month of pregnancy. A negative correlation was observed between plasma AMH concentrations in female calves and the daily milk yield, milk fat content, SNF content, milk protein content, and lactose content in their dams in the first month of pregnancy. This negative correlation persisted in the second month of pregnancy when the plasma AMH concentrations in female calves were inversely correlated with daily milk yield, milk fat content, SNF content, milk protein content, and lactose content in their dams. Furthermore, in the third month of pregnancy, plasma AMH concentrations in female calves were negatively correlated with daily milk yield, SNF content, milk protein content, and lactose content in their dams. Similar negative correlations were found in the fourth month of pregnancy, with the plasma AMH concentrations in female calves being negatively correlated with daily milk yield, milk fat content, SNF content, milk protein content, and lactose content in their dams. However, no correlation was observed between plasma AMH concentrations in female calves and the daily milk yield or milk component amounts in dams in the fifth month of pregnancy. At 6 mo of pregnancy, the only negative correlation observed was between the plasma AMH concentrations in female calves and the lactose content in their dams.

**Table 1:** Correlation coefficient (r) between plasma AMH concentrations of female calves and milk yield or milk composition of their dams (primiparous cows) during the first to sixth month of pregnancy

Item	Pregnancy month						SEM
	1 mo (n = 22)	2 mo (n = 22)	3 mo (n = 22)	4 mo (n = 22)	5 mo (n = 21)	6 mo (n = 20)	
Milk yield (kg/d)							
Average	29.9	28.0	26.2	25.0	23.2	20.9	0.72
r	−0.370	−0.422	−0.388	−0.473	−0.217	−0.260	
*P*-value	0.090	0.050	0.074	0.026	0.332	0.263	
Milk fat amount (kg/d)							
Average	1.12	0.94	0.98	0.97	0.93	0.87	0.026
r	−0.462	−0.411	−0.316	−0.465	−0.235	−0.062	
*P*-value	0.031	0.058	0.150	0.029	0.291	0.786	
SNF amount (kg/d)							
Average	2.70	2.39	2.39	2.27	2.14	1.94	0.065
r	−0.408	−0.425	−0.432	−0.508	−0.264	−0.289	
*P*-value	0.060	0.049	0.044	0.016	0.235	0.189	
Milk protein amount (kg/d)							
Average	1.02	0.91	0.93	0.88	0.84	0.78	0.025
r	−0.426	−0.454	−0.503	−0.576	−0.288	−0.224	
*P*-value	0.047	0.034	0.017	0.005	0.193	0.311	
Lactose amount (kg/d)							
Average	1.38	1.20	1.20	1.14	1.06	0.94	0.034
r	−0.386	−0.407	−0.412	−0.495	−0.251	−0.431	
*P*-value	0.076	0.060	0.057	0.019	0.259	0.045	

Table 2 presents the correlation coefficient between plasma AMH concentrations in female calves and the milk yield or milk composition of their dams (multiparous cows) during the first to sixth month of pregnancy. Negative correlations were observed in the fifth month of pregnancy between plasma AMH concentrations in female calves and the milk fat amount and milk protein amount in their dams. At 6 mo of pregnancy, the only negative correlation observed was between the plasma AMH concentrations in female calves and the daily milk yield in their dams. However, no correlation was determined between plasma AMH concentrations in female calves and the daily milk yield or milk components during the other months of pregnancy in their dams.

**Table 2:** Correlation coefficient (r) between plasma AMH concentrations of female calves and milk yield or milk composition of their dams (multiparous cows) during the first to sixth month of pregnancy

Item	Pregnancy month						SEM
	1 mo (n = 33)	2 mo (n = 32)	3 mo (n = 32)	4 mo (n = 33)	5 mo (n = 32)	6 mo (n = 31)	
Milk yield (kg/d)							
Average	32.2	31.8	30.1	28.7	26.9	24.6	0.71
r	−0.118	−0.207	−0.165	−0.164	−0.287	−0.297	
*P*-value	0.510	0.247	0.357	0.359	0.104	0.093	
Milk fat amount (kg/d)							
Average	1.21	1.12	1.14	1.10	1.08	0.99	0.029
r	−0.089	−0.201	−0.050	−0.155	−0.355	−0.241	
*P*-value	0.620	0.259	0.783	0.385	0.043	0.175	
SNF amount (kg/d)							
Average	2.87	2.73	2.63	2.60	2.46	2.27	0.065
r	−0.103	−0.222	−0.128	−0.207	−0.281	−0.255	
*P*-value	0.567	0.212	0.474	0.247	0.112	0.151	
Milk protein amount (kg/d)							
Average	1.09	1.05	1.02	1.02	0.99	0.91	0.025
r	−0.162	−0.238	−0.174	−0.251	−0.304	−0.287	
*P*-value	0.364	0.181	0.329	0.157	0.085	0.104	
Lactose amount (kg/d)							
Average	1.46	1.38	1.31	1.23	1.21	1.11	0.033
r	−0.101	−0.212	−0.118	−0.184	−0.265	−0.250	
*P*-value	0.572	0.235	0.510	0.302	0.135	0.160	

Although no research has investigated blood AMH concentrations in female calves immediately after birth, the blood AMH concentrations in Holstein heifers aged 12 to 15 mo have been reported to range from 53 to 1,224 pg/mL (Alward et al., 2021) and from 6 to 440 pg/mL in those aged 11 to 15 mo (Jimenez-Krassel et al., 2015). Furthermore, the blood AMH concentrations of adult cows during the breeding season, including Holstein, Jersey, and their crossbreeds, ranged from 10 to 3,198 pg/mL (Ribeiro et al., 2014). Postnatal blood AMH concentrations have been demonstrated to increase during the first 2 mo of age, decrease at 5 mo of age, and then stabilize at 8 to 9 mo of age, around puberty (Mossa et al., 2017). Because the concentrations naturally vary according to the month of age, it may not be meaningful to compare the results of the current study with those of earlier research. However, what these studies have in common is that the concentration range is wide, indicating large individual differences, and the histograms are skewed to the right (a distribution with a short left-hand tail and a large right-hand tail), as in this study. Souza et al. (2015) found strong positive correlations among AMH measurements taken from individual cows at different phases of the estrous cycle and mentioned the high repeatability of AMH measurements across different phases. Additionally, the pregnancy rate in dairy cows with high plasma AMH concentrations is higher than that in dairy cows with low and intermediate concentrations (Ribeiro et al., 2014). Therefore, although the present study did not investigate blood AMH concentrations during the growth period of the female calves examined in this study, this individual difference is likely to be maintained throughout life and reflects the future reproductive performance of the female calves.

Primiparous cows, which are still in the growth phase, allocate ingested nutrients not only for lactation, but also for vital maintenance, growth, body fat accumulation, and during pregnancy, placental and fetal development (Redmer et al., 2004; National Agriculture and Food Research Organization, 2017). In addition, during early pregnancy when milk production is still high, the number of primordial follicles in the fetus increases and reaches maximum on d 110 to 120 (Erickson, 1966; Tanaka et al., 2001). The primiparous cows in this study exhibited numerous negative correlations between the plasma AMH concentrations in their female calves immediately after birth and the daily milk yield and milk component amount from the first to the fourth month of pregnancy. This suggests that increased milk and milk component production in primiparous cows requires a higher energy intake, possibly leading to less nutrient distribution for placental and fetal development, thereby resulting in lower plasma AMH concentrations in female calves. Conversely, no correlation was observed between plasma AMH concentrations in multiparous cows and the daily milk yield or milk component amounts during the first to fourth month of pregnancy. Akbarinejad et al. (2018) found higher blood AMH concentrations in cows born from multiparous cows compared with those born from nulliparous and primiparous cows. They attributed this difference to differential nutritional partitioning in dairy cows of different parities and the presence of smaller uterine arteries with less blood flow in younger animals with fewer pregnancies than in multiparous animals (Akbarinejad et al., 2018). Moreover, high-producing dairy cows in early pregnancy may benefit from larger individual cotyledons during late pregnancy (Mashimo et al., 2023). This could contribute to providing similar nutrition to the fetus and facilitate the recovery of birth weight compared with low-producing dairy cows (Mashimo et al., 2023).

In this study, no significant difference was observed in the plasma AMH concentrations of female calves born from nulliparous heifers or primiparous cows and multiparous cows, despite the high concentrations in the plasma of these calves born from multiparous cows. This study yielded different results from those of Akbarinejad et al. (2018), who investigated blood AMH concentrations when the concentrations were relatively stable following the first calving. The smaller sample size in this study than that in Akbarinejad et al. (2018), or the analysis of AMH concentrations immediately after birth, for which there are still insufficient data in this study, could be the cause of the discrepancy in results. This discrepancy, however, could be explained by the fact that in multiparous cows, placenta and uterine artery development may help supplement the fetuses' nutritional supply and lessen the suppression of primordial follicle proliferation during the early stages of pregnancy (Akbarinejad et al., 2018; Mashimo et al., 2023). In any case, not much is known about AMH concentrations in female calves immediately after birth, and further research is required to address these gaps. Additionally, more research is needed to examine not only milk yield and composition but also factors such as diseases that affect the energy status of the dams to elucidate the factors that affect the number of primordial follicles in fetuses.

In conclusion, our results indicate individual variations in plasma AMH concentrations immediately after birth. Additionally, our findings suggest that both milk yield and milk components exert a strong influence on the plasma AMH concentrations immediately after birth in female calves of primiparous cows in the growth phase, whereas in multiparous cows, no such effect was observed. The results of this study highlight the importance of feeding management in high-producing pregnant primiparous cows, as their allocation of energy to growth and lactation may compromise the reproductive potential of their offspring. Improving the feeding management of primiparous cows to improve the fertility of their offspring may contribute to the successful reproduction of high-yielding dairy cows.
